# Injuries associated with arm wrestling: A narrative review

**DOI:** 10.1016/j.jcot.2021.04.010

**Published:** 2021-04-20

**Authors:** Darren Patrick Moloney, Iain Feeley, Andrew J. Hughes, Khalid Merghani, Eoin Sheehan, Muiris Kennedy

**Affiliations:** a University College Cork, College Road, Cork, Ireland; b Department of Trauma and Orthopaedics, Cork University Hospital, Wilton Road, Cork, Ireland; c Department of Trauma and Orthopaedics, Midland Regional Hospital Tullamore, Arden Road, Tullamore, Ireland

**Keywords:** Arm wrestling, Trauma, Humerus fracture, Medial epicondyle fracture, Skanderbeg, MeSH, Medical Subject Headings

## Abstract

**Objective:**

Arm wrestling is common sport amongst amateur enthusiasts. Multiple injuries are described as a result of the sport. The authors present a narrative review of the common injuries associated with the sport.

**Design:**

Systematic review with a critical appraisal of the literature and a narrative review of the injuries associated with arm wrestling.

**Data sources:**

Seven electronic databases were systematically searched using medical subject headings (MeSH) terms as follows. Arm wrestling, Indian Wrestling, Fractures, Injury, Ligament Injury with Boolean search terms “AND”. An extensive review of orthopaedic textbooks was also performed.

**Eligibility criteria for selecting studies:**

Inclusion criteria were publications which included patients who suffered bony or soft tissue injuries as a result of arm wrestling published in English language.

**Results:**

A total of 152 patients was seen across all studies. Spiral fractures of the distal third of the humerus are by far the most common injury reported in the setting of arm wrestling. The humerus fails due torsional and bending stresses. 23% were complicated by medial butterfly fragment and the incidence of radial nerve palsy was 23%. Fracture patterns differ in the skeletally immature arm wrestler, who show an increased incidence of medial humeral epicondyle fractures. We also report on the atypical fracture and soft tissue injury patterns that present.

## 1 Introduction

Arm-wrestling, also known as “Indian wrestling”, “Skanderbeg” and “wrist wrestling” can be traced back to ancient Egypt, documented with hieroglyphs in Beni Hasan tombs from the 21st Century Before the Common Era (BCE).^1^ Arm wrestling is a familiar sight in playgrounds, bars and amongst amateur enthusiasts. Formal competition began in the 1950’s. Competitive arm wrestling is sanctioned by the United States Arm-wrestling Federation (USAF) and the World Arm-wrestling Federation (WAF).^2^^,^^3^ It is divided into weight-based categories and by hand dominance.

Orthopaedic injuries as a result of wrestling may occur due to the enormous torsion that goes through the humerus and elbow joint during play. Humeral fractures as a direct result of arm wrestling are well described,^4^, ^5^, ^6^, ^7^, ^8^, ^9^, ^10^, ^11^, ^12^, ^13^, ^14^, ^15^, ^16^, ^17^, ^18^, ^19^, ^20^, ^21^, ^22^, ^23^, ^24^, ^25^ with a lower incidence of forearm and shoulder injuries reported.^26^, ^27^, ^28^, ^29^ Injury pattern differs with age.^18^^,^^21^^,^^30^, ^31^, ^32^

Humeral fractures as a result of arm wrestling tend to be described as rotational type spiral fractures of the distal humerus, with the radial nerve at risk as it traverses the spiral groove to the lateral intramuscular septum. Radial nerve palsy is reported in up to 22% of cases.^33^ This spiral fracture is also known as a Holstein Lewis fracture, eponymously named for Arthur Holstein and Gwylim Lewis who documented the fracture and associated radial nerve palsy secondary to interposition of the nerve in the fracture site in 1963.^34^^,^^35^

**Fig. 1:**
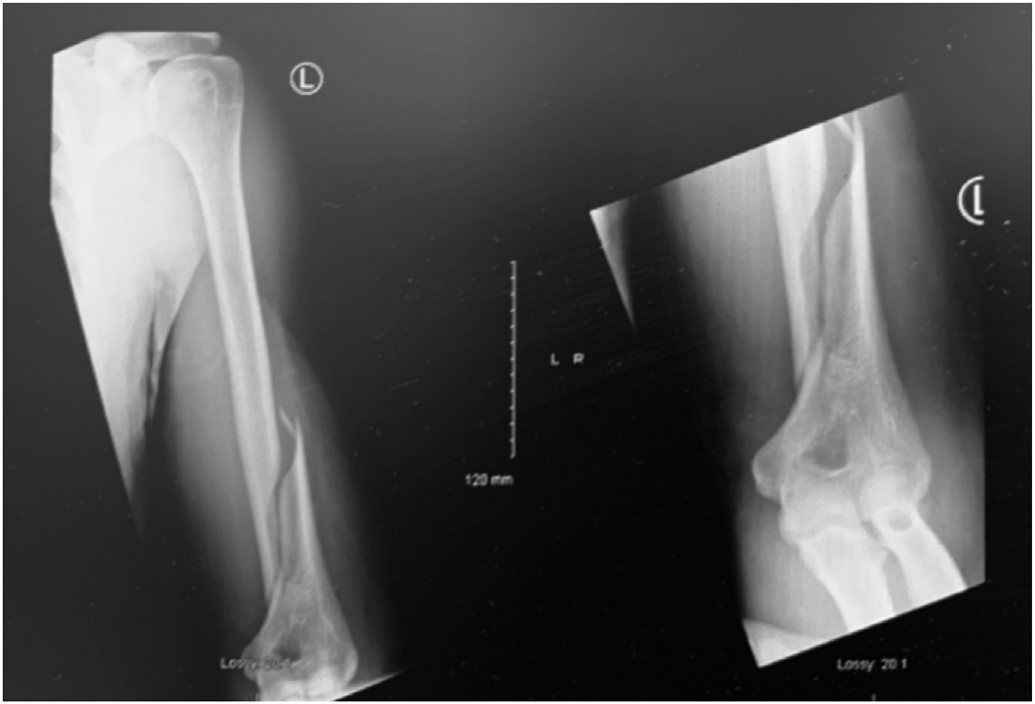
Holstein Lewis fracture in a 22-year-old arm wrestler.

The authors of this review were faced presentation and treatment of a distal humerus fracture which resulted from an amateur arm-wrestling match. The patient was a 22-year-old gentleman who was right hand dominant who suffered a left humeral shaft fracture as a result of left-handed arm wrestling. The fracture was classified as by the AO/OTA classification system as a 12A1.3, or the eponymously named Holstein-Lewis fracture (Fig. 1).^36^ The patient had no evidence of neuropraxia or vascular compromise at the time of presentation. The fracture was treated with an open reduction and internal fixation. This presentation led to a review of the published literature of injuries associated with arm wrestling.

## 2 Design

### 2.1 Data sources

**Fig. 2:**
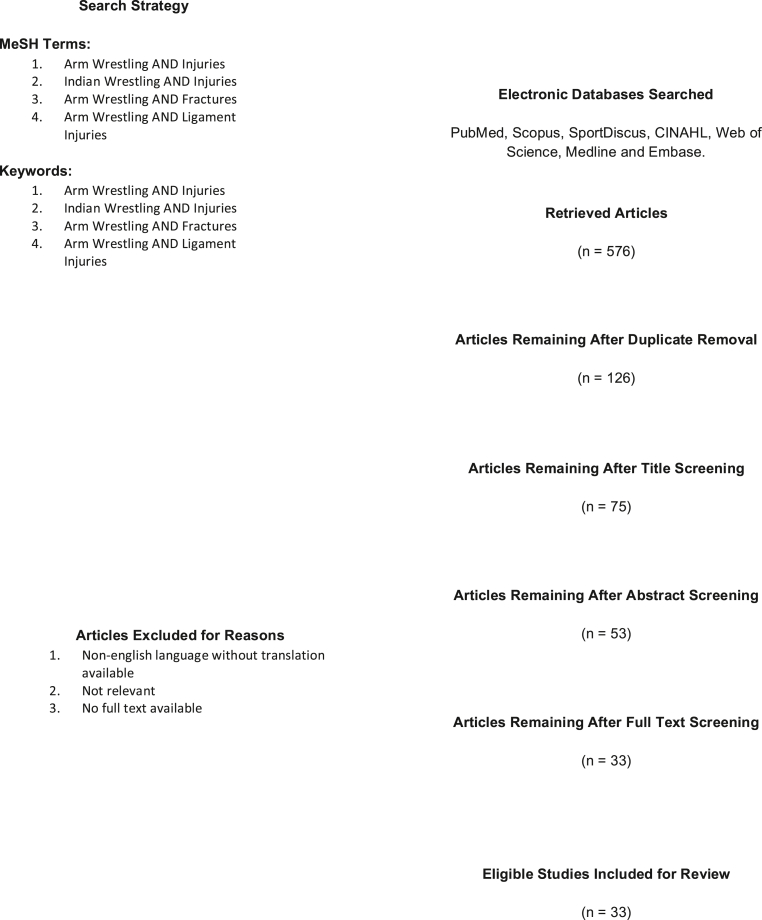
PRISMA flowchart.

The search was conducted through online search engines PubMed, Scopus, SportDiscus (1993–2020), Cumulative Index to Nursing and Allied Health Literature (CINAHL) (1996–2020), Web of Science, Medline (1996–2020), Embase (1947–2020). They were searched from their inception to January 18, 2020, using a preferred reporting items for systematic reviews and meta-analysis (PRISMA) compliant search strategy. The databases were systemically searched using four separate search terms: “Arm Wrestling” AND Injuries, “Arm Wrestling” AND Fractures, “Arm Wrestling” AND Fractures and “Arm Wrestling” AND “Ligament Injuries”. This combined approach allowed a comprehensive review of the literature. The keywords were assembled into Boolean search phrase using the phrase AND to allow for specific and accurate search. Our PRISMA flowchart is illustrated in Fig. 2.

### 2.2 Eligibility criteria

#### 2.2.1 Inclusion criteria

Studies were included if they met the following criteria.

1. Included patients who suffered bony or soft tissue injuries as a result of arm wrestling
2. Were published in English language
3. Not a compilation of previously published cases.

#### 2.2.2 Exclusion criteria

1. Studies were excluded which described injuries associated with other sports.

### 2.3 Data extraction and study appraisal

Articles meeting the inclusion criteria were imported into endnoteX9 (version 9.3.2) reference management software. They were screened for duplicates initially by author DM. They were then screened by title by author DM. The abstracts were then screened by two authors DM and IF. Abstracts identified by either author were included for full text assessment, and included in the systematic review by consensus. This process is illustrated in Fig. 2.

Data extraction was performed by DM, and tabulated on a Microsoft Excel spreadsheet (Excel™ version 16.16.18 Microsoft®, Redmond, WA) was used to document information on: study name, authors, year, study design, participant characteristics, sample size, injury type. Data was tabulated by injury type. i.e. humeral fractures, medial humeral epicondyle avulsion fractures, ligament injuries and atypical fractures.

The methodological quality of the studies were evaluated by principal investigator, DM and verified by a second author IF using a standardised tool for the critical evaluation of papers for quantitative research.

The search strategy found 576 articles. There were 33 studies included for review. The studies were of various designs including case reports, case series, biomechanical studies, radiological studies, retrospective observational studies and retrospective cohort studies.

### 2.4 Demographics

A total of 152 patients was recorded across all studies. Where gender was known there was a significant gender bias toward males 105/109 (96.3%). Patient types varied from competitive arm wrestlers, weightlifters and casual enthusiasts.

### 2.5 Humeral fractures

There were 108 humeral fractures across all studies. All patients had a spiral type of fracture. In cases where gender was recorded 66/70 were male (94.3%). Treatment means were not discussed in all papers. Patients were treated by both surgical and conservative measures. 50 patients underwent open reduction and internal fixation, and 47 patients were treated non operatively. No studies performed randomisation of their subjects into treatment groups. Results are summarised in Table 1.

**Table 1:** Humeral fractures as a result of arm wrestling.

Title	Author	Year	Journal	Study Design	Sample Size	Fracture Type	Treatment	Findings
Humeral fractures sustained during arm wrestling: A retrospective cohort analysis and review of the literature	Mayfield, C. K. Egol, K. A.	2018	Orthopedics	Retrospective Cohort Study	9 Arm Wrestlers 84 other MOI	Spiral fracture, distal one third humerus,	Conservative	Longer time to union for fractures in arm wrestlers
A spiral fracture of the humeral shaft due to arm wrestling	Demir, M. C. Ipek, A. B. Özdamar, Y. Karaca, M. A.	2018	Medicina dello Sport	Case Report	1 (1 M)	Spiral fracture, distal one third humerus,	ORIF	
Sudden elbow pain while arm wrestling	Maeder, B. Ngassom-Leumessi, E. Vauclair, F.	2017	Visual Journal of Emergency Medicine	Case Report	1 (1 M)	Spiral fracture, distal one third humerus, with medial butterfly fragment	Unknown	
Minimally invasive plate osteosynthesis by anterior approach: An alternative in distal humeral shaft fractures produced by arm wrestling	Sirbu, P. D. Berea, G. Asaftei, R. Tudor, R. Sova, R. Bodescu, A.	2016	Injury	Retrospective observational study	10	Distal Third of Humerus	Minimally invasive plate osteosynthesis	Operating Time - 66.5mins, radiation exposure 19.7 s, 9.6 weeks to radiological union, 100 Consant score for shoulder, 100 MEPI score for elbow
Humeral fracture in arm wrestling: Bone morphology as a permanent risk factor. Indications for safety measures in arm wrestling	Marks, W. Penkowski, M. Stasiak, M. Witkowski, Z. Dabrowski, T. Wieruszewski, J. Dudek, R. Lasek, J. Hauser, R.	2014	J. Sports Med. Phys. Fitness	Cadaveric Biomechanical Study	5 Cadaveric humeri	Spiral fracture, distal one third humerus,	Not Appilcable	In all three cases proximal end was in zone III and distal in zone II. The type of fracture was the spiral, external rotational type. The length of the fracture was 13%–42% of the entire length of the humerus, an average of 25 ± 7.1%.
Fractures of the humerus during arm wrestling	Bumbaširević, M. Ž Lešić, A. R. Andjelković, S. Z. Palibrk, T. D. Milutinović, S. M.	2014	Vojnosanitetski Pregled	Retrospective Cohort Study	6 (6 M)	Spiral fracture, distal one third humerus	3 - conservative, 3 open reduction and internal fixation	10 weeks to radiographic union, 16 weeks return to work, no significant difference between groups
Comminuted fracture with butterfly fragment of the humerus sustained during arm wrestling	Kim, H. S. Shin, Y. H. Kim, Y·W.	2013	Biomedical Research (India)	Case Report	1 (1 M)	Spiral fracture, distal one third humerus, with medial butterfly fragment	Conservative - immobilisation and long arm cast	
Humeral fractures by arm wrestling in adult: A biomechanical study	Pedrazzini, A. Pedrazzoni, M. De Filippo, M. Nicoletto, G. Govoni, R. Ceccarelli, F.	2012	Acta Biomedica de l’Ateneo Parmense	Cadaveric Biomechanical Study	5	Not appliacable	Not Appilcable	Lower bone mineral density in the distal third of the humerus. 40–60 MPa torsional strenght of humerus. Outside-inner diameter ratio most unfavoruable.
Radiological and biomechanical analysis of humeral fractures occurring during arm wrestling	Kruczynski, J. Jaszczur Nowicki, J. Topolinski, T. Srokowski, G. Manko, G. Chantsoulis, M. Frankowska, M. Frankowski, P.	2012	Med Sci Monit	Retrospective Cohort Study and Computer Aided Biomechanical Analysis	9 (8 M, 1F)	OTA-AO 12-B1 Spiral fracture, distal one third humerus, with medial butterfly fragment (n = 5). OTA-AO 12-A1 Spiral fracture, distal one third humerus (n = 4)	Open Reduction and Internal Fixation (1 AO plate, 5 LCP, 1 ZESPOL device, 1 EISIN wires)	30% incidence of radial nerve palsy. The maximum bone stress resulting from torsional loading which occurs during arm wrestling amounted to 60 MPa and was located 115 mm above the elbow on the medial - posterior side of the humeral.
Arm Wrestling Injuries - Report on 11 Cases with Different Injuries	Citak, M. Backhaus, M. Seybold, D. Muhr, G. Roetman, B.	2010	Sportverletzung-Sportschaden	Case Series	11	Spiral fracture, distal one third humerus (n = 6), spiral fracture midshaft humerus (n = 1)	Open Reduction and Internal Fixation	
Broken arm wrestler	Khashaba, A.	2000	British Journal of Sports Medicine	Case Report	1 (1 M)	Spiral fracture, distal one third humerus	Conservative	
Spiral fracture of the humerus caused by arm wrestling	Ahčan, U. Aleš, A. Završnik, J.	2000	European Journal of Trauma	Case Report	1 (1 M)	Spiral fracture, distal one third humerus	Open Reduction and Internal Fixation	
Arm wrestler’s fracture	Saab, M.	1999	Eur J Emerg Med	Case Report	1 (1 M)	Spiral fracture, distal one third humerus	Conservative - U-slab	
Humeral shaft fracture sustained during arm wrestling: Report on 30 cases and review of the literature	Ogawa, K. Ui, M.	1997	Journal of Trauma - Injury, Infection and Critical Care	Retrospective Observational Study	30 (28 M, 2 F)	Spiral fracture, distal one third humerus, 23% with medial butterfly fragment, 23% Radial nerve palsy	13 - Conservative - 100% union. 17 - Open reduction internal fixation - 100% union	Distal end of fracture, medial 24/30, Proximal end of fracture, posterior 13/30. Radial nerve palsy delayed return to work
Arm-wrestler’s injury: A report of thirteen cases	Moon, M. S. Moon, Y. W. Sihn, J. C. Kim, S. S. Sun, D. H. Kim, S·S.	1997	Journal of Orthopaedic Surgery	Retrospective Observational Study	13 (13 M)	5 - humeral shaft fractures	Unknown	
Fractures of the humerus in arm wrestling	Wagner de Barros, J. Oliveira, D. J.	1995	International Orthopedics	Case Series	2 (2 M)	Spiral fracture, distal one third humerus	1 U slab 1 hanging cast	
Fracture of humerus during use of an arm wrestling machine	Helm, R. H. Stuart, P.	1986	Br Med J (Clin Res Ed)	Case Report	1 (1 M)	Spiral fracture, distal one third humerus	U Slab immobilisation	10 weeks to radiographic union
Arm wrestler’s injury: report of seven cases	Moon, M. S. Kim, I. Han, I. H. Suh, K. H. Hwang, J. D.	1980	Clin Orthop Relat Res	Retrospective Observational Study	7	2 Spiral fracture of the humerus with medial butterfly fragments	U Slab immobilisation followed by hanging cast	
Fractures of the humerus in arm wrestlers	Heilbronner, D. M. Manoli, Ii A. Morawa, L. G.	1980	Clinical Orthopedics and Related Research	Case Series	2 (1F 1 M)	2 Spiral fracture of the humerus	1 Open reduction and internal fixation 1 hanging cast	
Arm wrestling fractures--a humerus twist	Whitaker, J. H.	1977	Am J Sports Med	Case Series	5 (5 M)	3 Spiral fracture of distal humerus 2 Spiral fracture of distal humerus with medial butterfly fragments	5 Hanging cast	1 radial nerve palsy resolved after 2 weeks
Fractures of the humerus from arm wrestling	Peace PK.	1977	Injury	Case Series	2 (2 M)	1 Spiral fracture of distal humerus 1 Spiral fracture of distal humerus with medial butterfly fragments	2 Hanging cast	
Fracture of the humerus from arm wrestling	Brismar, B. Spangen, L.	1975	Acta Orthop Scand	Case Series	2 (2 M)	2 Spiral fracture of distal humerus	1 Hanging cast 1 ORIF with radial nerve exploration	1 total radial nerve palsy

### 2.6 Medial epicondyle fractures

There were 5 studies which focused on medial epicondyle avulsion in the setting of arm wrestling. There were 35 patients. All patients were male. Treatment modality was not described for 7 of these cases. 15 were treated by collar and cuff immobilisation. 13 were treated by open reduction and k-wire fixation. Lokiec et al. reported a single case of medial epicondyle avulsion in an adult patient.^32^ Results are summarised in Table 2.

**Table 2:** Medial humeral epicondyle fractures as a result of arm wrestling.

Title	Author	Year	Journal	Study Design	Sample Size	Injury	Treatment	Findings
Arm-wrestler’s injury: A report of thirteen cases	Moon, M. S. Moon, Y. W. Sihn, J. C. Kim, S. S. Sun, D. H. Kim, S·S.	1997	Journal of Orthopaedic Surgery	Retrospective Observational Study	7 (7 M)	7 - medial humerus epicondyle fractures	Unknown	
Fracture-separation of the medial humeral epicondyle caused by arm wrestling	Ogawa, K. Ui, M.	1996	Journal of Trauma	Retrospective Observational Study	10	10 cases of medial humeral epicondyle fracture	2 conservative. 8 Open Reduction and K-wire fixation	Ulnar nerve paresis in one case, with ongoing symptoms at 8 years
Avulsion fracture of the medial epicondyle caused by arm wrestling	Nyska, M. Peiser, J. Lukiec, F. Katz, T. Liberman, N.	1992	American Journal of Sports Medicine	Retrospective Observational Study	8 (8 M)	8 Medial humeral epicondyle avulsion fractures	Collar and cuff Immobilisation	1 case of ulnar nerve paresis. 7 patients had 10° extension limitation at a year, 1 patient had 30° limitation at 1 year. 100% went on th ebony or fibrous union
Avulsion of the medial epicondyle of the humerus in arm wrestlers: a report of five cases and a review of the literature	Lokiec, F. Velkes, S. Engel, J.	1991	Injury	Case Series	5 (5 M)	5 Medial humeral epicondyle avulsion fractures	Collar and cuff Immobilisation	4 male teenagers, 1 39-year-old male
Arm wrestler’s injury: report of seven cases	Moon, M. S. Kim, I. Han, I. H. Suh, K. H. Hwang, J. D.	1980	Clin Orthop Relat Res	Retrospective Observational Study	5	5 Medial humeral epicondyle avulsion fractures	Open reduction and Internal fixation	

### 2.7 Rare fracture patterns and soft tissues injuries

Fracture patterns not included above are tabulated in Table 3. The unusual fracture patterns include an isolated radial shaft fracture, a scapular neck fracture and a radial neck fracture. One patient had extraarticular olecranon fracture. There were five soft issue injuries reported in the literature. Ligamentous and tendinous injuries are reported throughout the upper limb. There are two elbow injuries medial collateral ligament rupture. There are two shoulder injruies subscapularis and long head of biceps. There is a single documented case of ulnar collateral injury of the thumb as a result of arm wrestling. All patients were male. Results are summarised in Table 4.

**Table 3:** Atypical fractures as a result of arm wrestling.

5	Author	Year	Journal	Study Design	Sample Size	Injury	Treatment
Fracture of the scapular neck sustained in an arm-wrestling match	Considine, S. Hirpara, K. M. Hynes, D. E.	2014	Irish medical journal	Case Report	1	Extra-articular scapular neck fracture	Conservative - sling immobilisation
Arm Wrestling Injuries - Report on 11 Cases with Different Injuries	Citak, M. Backhaus, M. Seybold, D. Muhr, G. Roetman, B.	2010	Sportverletzung-Sportschaden	Case Series	11	Radial shaft fracture (n = 1)	Open reduction and Internal fixation
Olecranon fracture sustained in arm wrestling	Pasquina, P. F. O’Connor, F. G.	1999	Physician and Sportsmedicine	Case Report	1	Olecranon Fracture - circular-appearing fracture of the olecranon, which did not appear to be intra-articular.	Conservative - sling immobilisation
An Unusual Fracture in Arm Wrestling	Fallis, G. Ferguson, K. Malcolm, B.	1990	Phys Sportsmed	Case Report	1	Radial neck fracture	Collar and cuff Immobilisation

**Table 4:** Ligamentous and tendinous injuries as a result of arm wrestling.

Title	Author	Year	Journal	Study Design	Sample Size	Injury	Treatment	Findings
Use of sonography in assessing elbow medial collateral ligament injury after arm wrestling	Lee, Y. S. Chou, Y. H. Chiou, H. J. Lai, Y·C.	2014	J Chin Med Assoc	Case Report	1	Medial Collateral Ligament Rupture	Conservative	Increased ulnotrochlear joint space on dynamic ultrasonography, MCL tear evident
Proximal biceps rupture: Management of an unusual injury in an arm wrestler	Pratt, D. A. Tennent, T. D.	2007	British Journal of Sports Medicine	Case Report	1	Proximal Biseps tendon rupture	Long head of biceps tenodesis	
Anterior dislocation of the elbow in an arm wrestler	Torchia, M. E. DiGiovine, N. M.	1998	J Shoulder Elbow Surg	Case Report	1	Medial Collateral Ligament Rupture, Triceps tendon avulsion, flexor-pronator origin avulsion	MCL repair, triceps tendon repair, ulnar nerve exploration, flexor-pronator origin repair	
Rupture of the ulnar collateral ligament of the thumb in an arm wrestler	Faraj, A. A. Tang, D.	1998	Sports Exercise and Injury	Case Report	1	Ulnar collateral ligament of thumb MCPJ rupture		
Isolated rupture of the subscapularis tendon in an arm wrestler	Biondi, J. Bear, T. F.	1988	Orthopedics	Case Report	1	Rupture of subscapularis tendon		

## 3 Discussion

### 3.1 Biomechanical analysis of arm-wrestling

Spiral fractures of the distal third of the humerus are by far the most common injury reported in the setting of arm wrestling.^4^, ^5^, ^6^, ^7^^,^^9^, ^10^, ^11^, ^12^, ^13^, ^14^, ^15^, ^16^, ^17^, ^18^, ^19^, ^20^, ^21^, ^22^, ^23^, ^24^, ^25^^,^^30^ The underlying biomechanics of the injury have been the subject of some investigation with Brismar et al., in 1975 described the forces acting on the humerus during play. In the neutral position both players sit facing one another with their elbows on a flat surface attempting to overcome the opposing force of their rival.^25^ The shoulder is flexed at 45°. The humerus is subject to forces of internal rotation at the shoulder joint with the actions of pectoralis major, latissimus dorsi, subscapularis and teres major. The elbow is in fixed flexion with the biceps brachi, brachioradialis and brachialis undergoing isometric contraction. The wrist is initially in semi-supinated with the flexors and pronators undergoing isometric contraction. The humerus is a hollow cylinder is undergoing a bending moment, axial compression and torsional strain.

If we now examine the winning athlete. There is concentric muscle contraction around the shoulder joint causing progressive internal rotation. The elbow joint remains fixed in flexion with the muscles in isometric contraction. The wrist eventually flexes at the late stages of the match to end the bout, with the pronators and flexors undergoing concentric contraction.

The losing athlete in contrast undergoes eccentric contraction of the internal rotators surrounding the shoulder, with eventual extension of the of the elbow and wrist joint i.e. eccentric contraction of the elbow flexors, wrist flexors and pronators.

Taking the humerus in isolation one can see that there is an external rotation moment about which the competitor is trying to resist the external rotation force.^23^ The equation that relates to shear stress due to torque is as follows:

$$\tau =\frac{Tr}{I}$$

Stress is signified by *τ*, torque is signified by *T* and *r* is the distance from the axis of rotation to the point where the shear stress is calculated. I is the moment of inertia. The equation for the moment of inertia in a hollow cyclinder is as follows:

$$I=\;\frac{\pi \left(D^{4}-d^{4}\right)}{32}$$

*D* is the outside diameter of a hollow cylinder and *d* is the inner diameter of a hollow cylinder. To find the maximum shear stress in a hollow cylinder we must maximise r in the first equation thus substituting in the outside radius of the hollow cylinder i.e. *D/2.* We can also substitute for *I*. This results in the following expression:

$$\tau_{max}=\frac{16TD}{\pi \left(D^{4}-d^{4}\right)}$$

To find max shear stress we must find the portion of the cylinder which has the least favourable ratio between the inner diameter and outer diameter of the humerus. Pedrazzini at al examined cadaveric sections of 5 humeri.^11^ They showed lower bone mineral density in the distal third of the humerus. They also hypothesise that at the distal third of the humerus the ratio between the outer and inner diameter of the bone is less than in other areas of the bone, thus maximising the denominator in the above equation. This fact makes it most susceptible to fracture at this point due to shear stress. This hypothesis supports fracture patterns described in the literature.

Kruczynski et al. used commuted tomography of a right humeral bone to establish a virtual three-dimensional model of a humerus made from aluminium as it has a similar strength properties to human bone (Youngs modulus = 0.675 MPa, Poisson ratio v = 0.33). They found that stress was maximal at 115 mm above the elbow joint on the posteromedial aspect of the bone. Stress distribution is typical for torsional loading and the fracture line propagates at 45° to the long axis of the bone resulting in a spiral pattern fracture.^12^ It is noted however that this is considering that the strain is purely torsional however it is noted in reality there is also a bending moment created by the competitors humeral head stabilisers and shoulder adductors in response to the opposing force as well as axial compression of the humerus.

These biomechanical factors highlight the reasons why the humerus fails in a particular way in the setting of arm wrestling i.e. a spiral fracture at the distal third of the humerus with or without a butterfly fragment. Considering a pushing force of 20 kg (200 N), and a forearm 0.4 m long, there would be 80Nm of force acting upon the humerus. Kruczynski et al. calculated a force of 50–71Nm as causing fracture of the humerus.^12^

### 3.2 Humeral fractures

Brismar et al. Peace et al. and Whitaker et al. described the spiral fracture types in the mid to late 70s.,^23^, ^24^, ^25^ and the prescribed treatment modality was with a hanging cast in all but 1 reported case. The operative case had a concurrent radial nerve palsy. Heilbronner et al. and Moon et al. both described these fracture patterns in the 1980’s treating them by conservative means with the exception of one ORIF due to failure of the hanging cast, reportedly secondary to the patient abdominal adiposity.^21^^,^^22^ The majority of cases are documented in small case reports and case series.^5^^,^^6^^,^^10^^,^^13^, ^14^, ^15^, ^16^^,^^19^, ^20^, ^21^, ^22^, ^23^, ^24^, ^25^

Ogawa et al. performed a retrospective observational study examining 30 cases of humeral fracture secondary to arm wrestling.^17^ This group treated 17/30 non operatively with 100% union rate, 13 by ORIF with 100% union rate. They found 23% were complicated by medial butterfly fragment and the incidence of radial nerve palsy was 23%. Similar figures as are seen traditionally in Holstein-Lewis type, OTA 12A1.3 fractures.^33^ Ogawa et al. also developed an anatomical description of humeral zones from the insertion of the supraspinatus tendon to the line connecting the medial and lateral epicondyles, divided into five zones of equal length, numbered I–V from distal to proximal. They found that the fractures arose in zone I-III 90% of the time.

Mayfield et al. performed a retrospective cohort study in 2018 which looked at a group of humeral shaft fracture and analysed the results of 9 which occurred as a result of arm wrestling compared to 84 with an unspecified mechanism of injury. Treatment was non operative and the found significantly longer time to union in arm wrestlers.^4^

Sirbu et al. showed that minimally invasive plate osteosynthesis an effective treatment method for fractures in an arm wrestler, with good outcomes in terms of union rates, radiation exposure and elbow and shoulder patient reported outcomes measures.^7^

### 3.3 Medial epicondyle avulsion fracture

Considering the flexion of the wrist joint and protonation of the forearm in the winning competitor of the match vs eccentric contraction of the same muscle groups in losing opponent in the final stages, medial humeral epicondyle fractures are commonly seen in the skeletally immature patient.^18^^,^^21^^,^^30^, ^31^, ^32^ Due to the close proximity of the ulnar nerve to this area ulnar nerve paresis is reported at approximately 10–12.5%.^30^^,^^31^ One case of medial epicondyle avulsion is reported in an adult patient.^32^

### 3.4 Atypical fractures in arm-wrestling

A number of rare injuries have been reported as a result of arm wrestling. Considine et al. report a fracture of the scapular neck.^26^ Pasquina et al. report an extraarticular undisplaced olecranon fracture which was treated conservatively.^27^ Citak et al. described a spiral fracture of the mid radial shaft in fitting with the torque applied to the radius by the pronators during an arm wrestling match.^13^ A radial neck fracture is also described in the setting of arm wrestling.^28^

### 3.5 Soft tissue injuries

Medial collateral ligament injuries have been reported in the setting of arm-wrestling and dynamic ultrasonography is a useful tool for assessing joint space widening and medial collateral ligament (MCL) rupture.^37^ Long head of biceps has been reported as ruptured in the setting of arm wrestling. This is perhaps due to the isometric contraction of the biceps in an attempt to maintain fixed flexion at the elbow joint.^38^ The long head of biceps is known to show increased electromyographic activation in a position of shoulder flexion employed in arm wrestling.^39^ Ulnar collateral ligament rupture or “skier’s thumb” or “gamekeeper’s thumb” has been reported secondary to arm-wrestling.^40^ Isolated rupture of the subscapularis tendon has also been reported likely due to the massive internal rotation moment at the shoulder joint.^29^ Torchia et al. report anterior elbow dislocation.^41^ This rather catastrophic injury was associated with gross instability of the elbow joint due to triceps tendon avulsion, flexor-pronator insertion avulsion and medial collateral ligament rupture. The ulnar nerve also was found to have subluxated anterior to the medial epicondyle at the time of surgery.

Torsional stresses on the humerus may result in fractures in both skeletally mature and immature participants. There is a high rate of nerve palsy and may require significant rehabilitation thereafter. The authors recommend that arm wrestling should be approached with caution. Arm wrestling should be avoided in the skeletally immature. Weight based categories should be stratified in a competition setting. Participants should be appropriately conditioned prior to partaking to avoid soft tissue injuries. Competition athletes also practice appropriate technique. The position termed the “broken arm position” is avoided which minimises torsional stresses on the humerus during arm wrestling. Casual enthusiasts and young people may not practice these appropriate techniques making them susceptible to injury.

## 4 Conclusion

Since the early seventies it has been recognised that humeral fractures as a result of arm wrestling follow a typical pattern. Given the biomechanical considerations of the of the forces acting on the humerus it is an intuitive pattern of injury.

It has been shown in recent biomechanical studies why the distal third of the humerus is prone to the injury, due the unfavourable ratio of inner to outer diameter of the bone at this level which has been supported by computer modelled theories.

Rarer injuries are also reported in the literature therefore one must be wary of a patient presenting with this mechanism of injury., and consider injuries that are not typically associated with arm wrestling such as forearm injuries, shoulder injuries, soft tissue injuries and even hand injuries.

## Abbreviations

BCE: Before The Common Era
CINAHL: Cumulative Index To Nursing And Allied Health Literature
D: Outer Diameter
d: Inner Diameter
I: Intertia
kg: Kilogram
m: Meter
MCL: Medial Collateral Ligament
MeSH: Medical Subject Headings
mm: Millimetre
MPa: Megapascals
N: Newton
Nm: Newton Meter
OTA: Orthopaedic Trauma Association
r: Radius
T: Torque
USAF: United States Arm-Wrestling Federation () And The ()
WAF: World Arm-Wrestling Federation
*τ*: Stress
τmax: Maximum Stress
